# Annotations

**Published:** 1904-05-14

**Authors:** 


### ANNOTATIONS.

### "Academy" Headache.

"Academy" or "sightseers'" headache is a not uncommon experience, and some recent observations of Mr. Simeon Snell appear to throw some light on its causation. Allowing that in certain instances astigmatism or other form of ametropia is a factor in its production, he yet argues that there are other influencing conditions. In support of this he quotes the experience of a lady who always suffered from severe headache after visiting the theatre when she had occupied a seat in the pit, but was free from such disturbance when she sat in the dress circle. In the first position, it is pointed out, sustained action of the elevator muscles of the eyeballs is required in order to see the stage, and this is exactly the action which is required in studying a collection of pictures, and more especially in looking at those which are hung "above the line." The same experience has been noted by Mr. Snell in the case of cyclists, particularly of those who lean over the handle-bars with the head lowered, and who, therefore, in looking ahead keep up a strain on the muscles which turn the eyeballs upwards. A further argument is found in the experience of miners' nystagmus, which it is suggested is due to weariness of the elevator muscles of the eyeballs caused by the constrained position in which the miner works. The same thing has been noted in compositors, plate-layers, and many other occupations demanding prolonged use of the elevators. Whilst, however, in some of these nystagmus develops, in others the complaint is of aching and discomfort in the eyes. There can be no doubt of the physiological fact that lateral movement of the eyeballs is much more easy and requires less muscular effort than movement in a vertical plane, and for this there is an obvious utilitarian explanation. A perfect square, for example, looks longer from above downwards than it does from side to side, and landscape painters habitually exaggerate the height of high objects in order to produce a "natural" impression. All this goes to show that muscular strain is involved in sustained upward movement of the eyeballs, and this may well result in a sense of weariness or more pronounced discomfort.

### The Constituents of Patent Medicines.

An American contemporary again takes up the subject of the consumption of patent medicines. It publishes an analysis of no fewer than thirty-six "tonics," "bitters," and "sarsaparillas," and shows that these compounds contain alcohol in the proportion of from 13 to 47 per cent. Besides alcohol in amounts which make these mixtures, as the editor says, "stronger than whisky, far stronger than sherry or port, with claret and champagne way behind," many of these so-called tonics contain dangerous drugs. One "Favourite Prescription" which a pregnant woman was recommended by a friend to take, to relieve the ordinary discomforts of her condition, contained digitalis and opium, in addition to 17 per cent. of alcohol. Naturally these things are consumed by people who will not consult a doctor. They save a few shillings in medical advice, and injure their health, and even risk the contracting of the most insidious kind of drunkenness, with less hesitation than they would show in sampling a new soap. Although the habit of drug-drinking is not so rampant here as in America, it is popular enough. Women of the lower and lower middle classes are perhaps the most given to the habit, although it is confined to no special section of the community. They take these concoctions and they recommend them to each other, and if one should venture to advise care in their consumption the reply is often that they must be all right, because they have the Government stamp upon them. That this means nothing more than the payment of a tax they do not understand. Much would be done to stay the evil if it were made compulsory to inform purchasers what the various proprietary remedies contain. For there is small doubt that a knowledge of their composition would lead to a speedy diminution of the consumption of many of the popular nostrums of the day.

### The Relationship of the Rat to Human Plague.

In a report on plague in Hong Kong during 1903, just issued by Dr. Pearse, acting medical officer of health for the colony, it is stated that during the epidemic last year a systematic collection of rats was made throughout Victoria and Kowloon, the rats being afterwards bacteriologically examined at the public mortuary. A price of five cents a rat was paid throughout the year, and between ninety and a hundred thousand rats were caught. Unfortunately, it has not been possible to make any definite statement regarding the influence of rat plague on human plague, owing to the practical certainty that a great number of the rats collected by the coolies were imported into the city for the sake of the bonus on the dead bodies of the rodents. Dr. Pearse was, however, able to draw up curves showing the rise and fall in the percentage of infected rats among those examined in the public mortuary, and the weekly number of human plague cases. It appears from these that both rise to their highest points about the same time. The first notable rise in rat infection was in the sixth and seventh weeks of the year, and human plague made a sudden rise in the sixth week, though falling again the seventh. The ninth, tenth, twelfth and thirteenth weeks were marked by a rise both in rat infection and in human plague; and there was a more notable rise in human plague in the fourteenth week. In that week there was a drop in rat infection, and also in the fifteenth week, when a drop likewise took place in human plague. After this the figures do not correspond so closely, the human curve reaching its maximum with a rapid jump in the twenty-first week, while the rat infection was not at its highest until the twenty-fourth week. The human curve dropped far more rapidly towards the end of the epidemic; and the final conclusion drawn by Dr. Pearse is that there were factors in the shaping of the epidemic which affected the rats earlier, and of which the influence passed off later from rats than in the case of human beings. The fact that, owing to the continued prevalence of the disease in the colony, the first shipment of Chinese from Hong Kong to the Transvaal has been postponed emphasises the importance of all investigations bearing upon the epidemic.

